# Reversely immortalized mouse salivary gland cells presented a promising metabolic and fibrotic response upon BMP9/*Gdf2* stimulation

**DOI:** 10.1186/s11658-022-00333-9

**Published:** 2022-06-11

**Authors:** Wenping Luo, Panpan Liang, Tianyu Zhao, Qianyu Cheng, Huikai Liu, Liwen He, Linghuan Zhang, Bo Huang, Yuxin Zhang, Tongchuan He, Deqin Yang

**Affiliations:** 1 Chongqing Key Laboratory of Oral Diseases and Biomedical Sciences, 426 Songshi North Road, Yubei District, Chongqing, 401147 China; 2 Department of Surgery, Laboratory of Craniofacial Biology and Development, Section of Plastic Surgery, The University of Chicago Medical Center, 5841 South Maryland Avenue MC6035, Chicago, IL 60637 USA; 3 Stomatological Hospital of Chongqing Medical University, 426 Songshi North Road, Yubei District, Chongqing, 401147 China; 4 Chongqing Municipal Key Laboratory of Oral Biomedical Engineering of Higher Education, 426 Songshi North Road, Yubei District, Chongqing, 401147 China; 5 Stem Cell Biology and Therapy Laboratory, Ministry of Education Key Laboratory of Child Development and Disorders, The Children’s Hospital of Chongqing Medical University, Chongqing, 400014 China; 6 Jiangxi Province Key Laboratory of Laboratory Medicine, Department of Clinical Laboratory, The Second Affiliated Hospital of Nanchang University, No.1 Min De Road, Nanchang, 330006 China

**Keywords:** Submandibular gland, Sublingual gland, Immortalization, Metabolism, Fibrosis, BMP9/*Gdf2*

## Abstract

The submandibular gland (SMG) and the sublingual gland (SLG) are two of the three major salivary glands in mammals. In mice, they are adjacent to each other and open into the oral cavity, producing saliva to lubricate the mouth and aid in food digestion. Though salivary gland dysfunction accompanied with fibrosis and metabolic disturbance is common in clinic, in-depth mechanistic research is lacking. Currently, research on how to rescue salivary function is challenging, as it must resort to using terminally differentiated acinar cells or precursor acinar cells with unknown differentiation. In this study, we established reversely immortalized mouse primary SMG cells (iSMGCs) and SLG cells (iSLGCs) on the first postnatal day (P0). The iSMGCs and iSLGCs grew well, exhibited many salivary gland characteristics, and retained the metabolism-related genes derived from the original tissue as demonstrated using transcriptome sequencing (RNA-seq) analysis. The Kyoto Encyclopedia of Genes and Genomes (KEGG) pathways of these two cell lines, which overlapped with those of the SMG and SLG, were enriched in cysteine and methionine metabolism. Furthermore, we investigated the role of bone morphogenetic protein 9 (BMP9), also known as growth differentiation factor 2(*Gdf2*), on metabolic and fibrotic functions in the SMG and SLG. We demonstrated that iSMGCs and iSLGCs presented promising adipogenic and fibrotic responses upon BMP9/*Gdf2* stimulation. Thus, our findings indicate that iSMGCs and iSLGCs faithfully reproduce characteristics of SMG and SLG cells and present a promising prospect for use in future study of salivary gland metabolism and fibrosis upon BMP9/*Gdf2* stimulation.

**Supplementary Information:**

The online version contains supplementary material available at 10.1186/s11658-022-00333-9.

## Introduction

The three major salivary glands in mammals are the submandibular gland (SMG), the sublingual gland (SLG), and the parotid gland (PG). These glands are designed to produce and secrete saliva [1]. Genetic evidence has shown that SMG and SLG have different developmental origins compared with PG [2]. In terms of functionality, unlike PG, SLG and SMG are mainly responsive to resting saliva flow. Furthermore, they are composed of mucous and serous acini and produce a mucin-containing secretion, while the PG secretory acini are mostly pure serous acini and produce amylase [3, 4].

SMG and SLG share a similar developmental pattern of branching morphogenesis, and adjacent location in mice [5–7]. The mesenchyme provides critical cues for oral epithelium thickening to form a placode at embryonic day 11 (E11). The primary bud structure of SMG forms at E12 and then undergoes enlargement, cleft formation, branching, and differentiation and then a tree-like structure appears comprising the terminal secretory units, acini, and ducts at E15. SLG morphogenesis progress begins at E13. Recent studies have improved our understanding of stem/progenitor cells and branching morphogenesis of the salivary gland, including the role of fibroblast growth factor (FGF) and Wnt/β-catenin signals, especially in SMG [8–10]. Notably, the key functions of the salivary glands are to produce saliva to aid in food lubrication, digestion, taste, and even immunity. As knowledge about the metabolic function of salivary glands is limited, identification of the relevant molecular events is critical. It has been reported that SLG and SMG probably display differential activation and secretion [1]. Limitations associated with the infeasibility of collecting highly purified SLG and SMG secretions has hindered further research on the metabolic function of these two glands.

Gene and stem cell therapy provide a prospect for the regeneration and repair of salivary gland fibrosis [11–13]. It is well established that duct-calculus-induced salivary gland fibrosis leads to xerostomia and metabolic disorder, but effective therapy is lacking. At least 14 Bmp types have been identified in humans and rodents [14, 15]. Bone morphogenetic protein 9 (BMP9, also known as *Gdf2*), a member of the transforming growth factor (TGF)-β superfamily, is mainly produced by the liver. In our previous study, we reported that BMP9 is one of the most potent BMPs in inducing osteogenic/odontogenic differentiation of mesenchymal stem cells (MSCs) [16–18]. Furthermore, it has been reported that BMP9 plays a critical role in hepatic glucose production, lipid metabolism, and fibrosis, including lung, liver, and cardiac fibrosis [19–21]. Increased expression of the TGF-β signaling pathway contributes to fibrogenesis and fibrosis resolution of SMG [22]. To date, few studies have explored the role of BMP9 in metabolic and fibrotic functions in SLG and SMG.

Immortalized normal but not neoplastic salivary gland cells would provide a stable cell source for cellular studies. As early as 1996, Laoide et al. reported the establishment of an immortalized submandibular gland ductal cell line derived from a 12-day-old or 22-day-old male transgenic mouse harboring the large T antigen of SV40 [23]. This system containing the constitutive adenovirus E1A promoter and the polyoma large T (PyLT) antigen of the polyoma virus leads to low-level gene expression of SV40 in a wide variety of tissues. In this study, we aimed to isolate mouse primary SMG cells (SMGCs) and SLG cells (SLGCs) at P0 on the basis of differential gene analysis using transcriptome sequencing (RNA-seq).

To overcome the practical difficulties associated with cell culture, we sought to establish reversely immortalized SMG cells (iSMGCs) and SLG cells (iSLGCs) using the SV40 T antigen flanked with flippase recognition target (FRT) sites. We successfully established immortalized iSMGCs and iSLGCs that exhibit partial salivary gland characteristics without the risk of tumorigenesis. We further demonstrated that iSMGCs and iSLGCs present promising adipogenic and fibrotic responses upon BMP9/Gdf2 stimulation. Thus, iSMGCs and iSLGCs can be used to study the metabolic and fibrotic functions of salivary glands in the future.

## Materials and methods

### Cell culture and chemicals

HEK-293 cell lines were purchased from ATCC (Manassas, VA, USA) and its derivative line 293pTP cells, and 293 Phoenix Ampho (293PA) cells were maintained in complete Dulbecco’s modified Eagle’s medium (DMEM) containing 10% fetal bovine serum (FBS; Hyclone, CA, USA), 100 units of penicillin, and 100 mg of streptomycin at 37 °C in 5% carbon dioxide (CO_2_). Recombinant adenoviruses expressing FLP recombinase, BMP9/*Gdf2*, and GFP were generated and amplified in 293pTP or HEK-293 cells. Retroviral vector SSR#41 expressing SV40 T antigen flanked with the FRT sites was packaged in 293PA cells. Unless indicated otherwise, all chemicals were purchased from Sigma-Aldrich (St. Louis, MO, USA) or Thermo Fisher Scientific (Pittsburgh, PA, USA).

### Isolation and immortalization of SMGCs (iSMGCs) and SLGCs (iSLGCs) from postnatal submandibular gland and sublingual gland on P0

The ethics committee of the Affiliated Hospital of Stomatology of Chongqing Medical University approved this study (grant no. 2021062). The “Guidelines for Ethical Review of Laboratory Animal Welfare” (GB/T35892-2018, China) was applied at our institution. In this study, all experiments were performed in accordance with the Basel Declaration and followed the “3R” principles of treating experimental animals: reduction, replacement, and refinement. Eight submandibular salivary glands (SMGs) were promptly removed from four newborn C57BL/6 N mice, which were euthanized using CO_2_. Next, SMGs were cut into 1 mm^3^ pieces, placed in a 35 mm dish with complete DMEM, and incubated for 72 h at 37 °C. Expansion cells were named “SMGCs” or “SLGCs.” The cells were suspended in DMEM complete medium containing 10% FBS, 100 units of penicillin, and 100 mg of streptomycin.

To establish the immortalized mouse submandibular gland cells (iSMGCs) and sublingual gland cells (iSLGCs), retrovirus SSR#41 was used as previously described [24, 25]. The SSR#41 retrovirus was packaged with 293PA cells. Briefly, 10 μg SSR#41 vector and 10 μg pCL-Ampho vector were cotransfected into 293 PA cells with 70% density for 4–6 h. After packaging for 36 h, the medium supernatant was collected four times at 12 h intervals. The collected medium supernatant was made free of cellular debris via centrifugation and a 0.45 μm membrane filter (SLHV033RB, Merck, Kenilworth, USA). Next, the passage of SMGCs or SLGCs was seeded in 25 cm^2^ flasks at 30–40% density, and then infected with SSR#41 retrovirus four times at 6–8 h intervals. The iSMGCs or iSLGCs were collected by adding hygromycin B (0.4 mg/ml) to the DMEM medium containing 2% FBS twice within 3–5 days, as previously described [24, 25].

### Generation and amplification of recombinant adenoviruses expressing BMP9, flippase (FLP), and GFP

Recombinant adenoviruses were generated using AdEasy technology as previously described [26, 27]. Ad-BMP9, Ad-FLP, and Ad-GFP were kindly provided by Professor Tong-Chuan HE (University of Chicago, Chicago, USA). Analogous adenovirus expressing GFP (Ad-GFP) alone was used as the control. The recombinant adenovirus coding regions of BMP9 (Ad-BMP9) and FLP (Ad-FLP) were generated and amplified in HEK-293 or 293pTP cells. Briefly, 70–80% of the 293 cells were replanted in a 100 mm dish for 4–6 h. After amplification for 48 h, cells and medium were harvested and centrifuged for approximately 10 min at 500*g* and 4 °C. Adenoviruses were released from cells through four freeze–thaw–vortex cycles, and then viral lysates were centrifuged at 500*g* and 4 °C to pellet the cell debris.

### RNA isolation, cDNA library construction, sequencing, and data analysis

Total tissue RNA of SMG and SLG on P0, and cell RNA of iSMGCs and iSLGCs, was extracted by Trizol reagent (15596018, Invitrogen, Carlsbad, CA, USA) following the manufacturer’s recommendations [28]. Enrichment of mRNA, fragmentation, reverse transcription, library construction, Hi-seq, and data analysis were performed by Genergy Biotechnology (Shanghai, China). Sequencing was performed on an Illumina HiSeq 2500 sequencer. The levels of gene expression were quantified using RSEM software. Differentially expressed genes (DEGs) were identified via the tool Cuffdiff. Genes with one or more fold changes in expression were selected, and *P* < 0.05 was considered statistically significant. The statistical enrichment of DEGs in KEGG pathways was compared with the whole genome background.

### RNA isolation and semi-quantitative RT-PCR (qPCR)

Total tissue and cell RNA was isolated using Trizol reagent (15596018, Invitrogen, Carlsbad, CA, USA) and used to generate cDNA templates via a reverse-transcription reaction kit (18090050, Invitrogen, Carlsbad, CA, USA). The cDNA products were used as PCR templates, as described previously [29]. PCR primers were designed using Primer 3.0 and are presented in Table 1. For qPCR analysis, SYBR Green-based qPCR analysis was conducted using the ABI Prism 7500 Real-Time PCR System (Applied Biosystems, Foster City, CA, USA). The qPCR reactions were completed in triplicate. All samples were normalized to GAPDH expression.

**Table 1:** Primer sequences

Primer name	Forward	Reverse
Gapdh Mus	5′-GGCTGCCCAGAACATCAT-3′	5′-CGGACACATTGGGGGTAG-3′
SV40 T Mus	5′-GGTGGGTTAAAGGAGCATGA-3′	5′-TAGTGGCTGGGCTGTTCTTT-3′
Sail Mus	5′-CTTGTGTCTGCACGACCTGT-3′	5′-AGGAGAATGGCTTCTCACCA-3′
Id1 Mus	5′-GCGAGGTGGTACTTGGTCTG-3′	5′-CTCCTTGAGGCGTGAGTAGC-3′
Ctgf Mus	5′-AAGGACCGCACAGCAGTT-3′	5′-AACAGGCGCTCCACTCTG-3′
BmprII Mus	5′-AGCGTCACAAGCCTGTCC-3′	5′-TTTGTGGCGTGCAAATGT-3′
Smad1 Mus	5′-AAGCTGTGGACGCTTTGG-3′	5′-ATCCAGGGAGCGAGGAAT-3′
Smad5 Mus	5′-TTCCATCCCCTGCCAGTA-3′	5′-GCCTCTCGGTGCTCTCTG-3′
Smad8 Mus	5′-ACCAGGAGGCACATTGGA-3′	5′-AGCCGTGCTGGTAGTTGC-3′
Pparγ2 Mus	5′-TTTTCAAGGGTGCCAGTTTC-3′	5′-AATCCTTGGCCCTCTGAGAT-3′
Col1a1 Mus	5′-GAGCGGAGAGTACTGGATCG-3′	5′-GCTTCTTTTCCTTGGGGTTC-3′
Bmp9 Mus	5′-TGAGTCCCATCTCCATCCTC-3′	5′-ACCCACCAGACACAAGAAGG-3′

### Immunofluorescence staining

Immunofluorescence staining was performed as described previously [30]. Briefly, cells were fixed with methanol or paraffin sections with sodium citrate for antigen retrieval, and then permeabilized with 0.1% Triton X-100, and blocked with 10% Block Aid (B10710, Invitrogen). Next, they were incubated with a primary antibody: AQP5 (1:400, pa5-97290, Thermo Fisher Scientific, Pittsburgh, PA, USA), α-SMA (1:200, ab124964, Abcam, Cambridge, UK), KRT19 (1:200, ab52625, Abcam, Cambridge, UK), and Vimentin (1:200, ab92547, Abcam, Cambridge, UK). After being washed, cells were incubated with 2 µg/ml donkey anti-rabbit IgG (H + L) highly cross-adsorbed secondary antibody, Alexa Fluor 488 (A-21206, Thermo Fisher Scientific, Pittsburgh, PA, USA). Nuclei were counterstained with DAPI (C1002, Beyotime, Shanghai, China). Stains were imaged under a laser scanning confocal microscope. Stains without primary antibodies or with control IgG were used as negative controls.

### Flow cytometry analysis of intracellular antigens

Intracellular antigens were analyzed via flow cytometry (FCM) according to the following protocol (BD Pharmingen, San Diego, CA, USA) [29–31]. Briefly, cells were harvested and fixed with formaldehyde to stabilize the cell membrane, and then permeabilized with detergent or alcohol to allow antibodies against intracellular antigens access to stain intracellularly. After being washed, cells were incubated with primary antibody: AQP5 (1:50, bs-1554R-AF647, Bioss, Beijing, China), α-SMA (1:200, ab124964, Abcam, Cambridge, UK), Krt19(1:200, ab52625, Abcam, Cambridge, UK), and Vimentin (1:200, ab92547, Abcam, Cambridge, UK). Next, cells were incubated with donkey anti-rabbit IgG (H + L) highly cross-adsorbed secondary antibody, Alexa Fluor 488 (A-21206, Thermo Fisher Scientific, Pittsburgh, PA, USA). All intracellular staining had to be completed in the presence of the permeabilization buffer and immediately detected by flow cytometry (BD Influx, CA, USA). Data were analyzed using Sortware software (1.0.0.650).

### Statistical analysis

The quantitative assays are presented as mean ± standard deviation (SD) and were performed in triplicate and/or repeated a minimum of three times. Statistical significance was determined via one-way analysis of variance (ANOVA) and Student’s *t*-test. All statistical analyses were conducted using SPSS 18.0 statistical software. The level of statistical significance was set at *P* < 0.05.

## Results

### SMG and SLG development and marker expression on P0

**Fig. 1:**
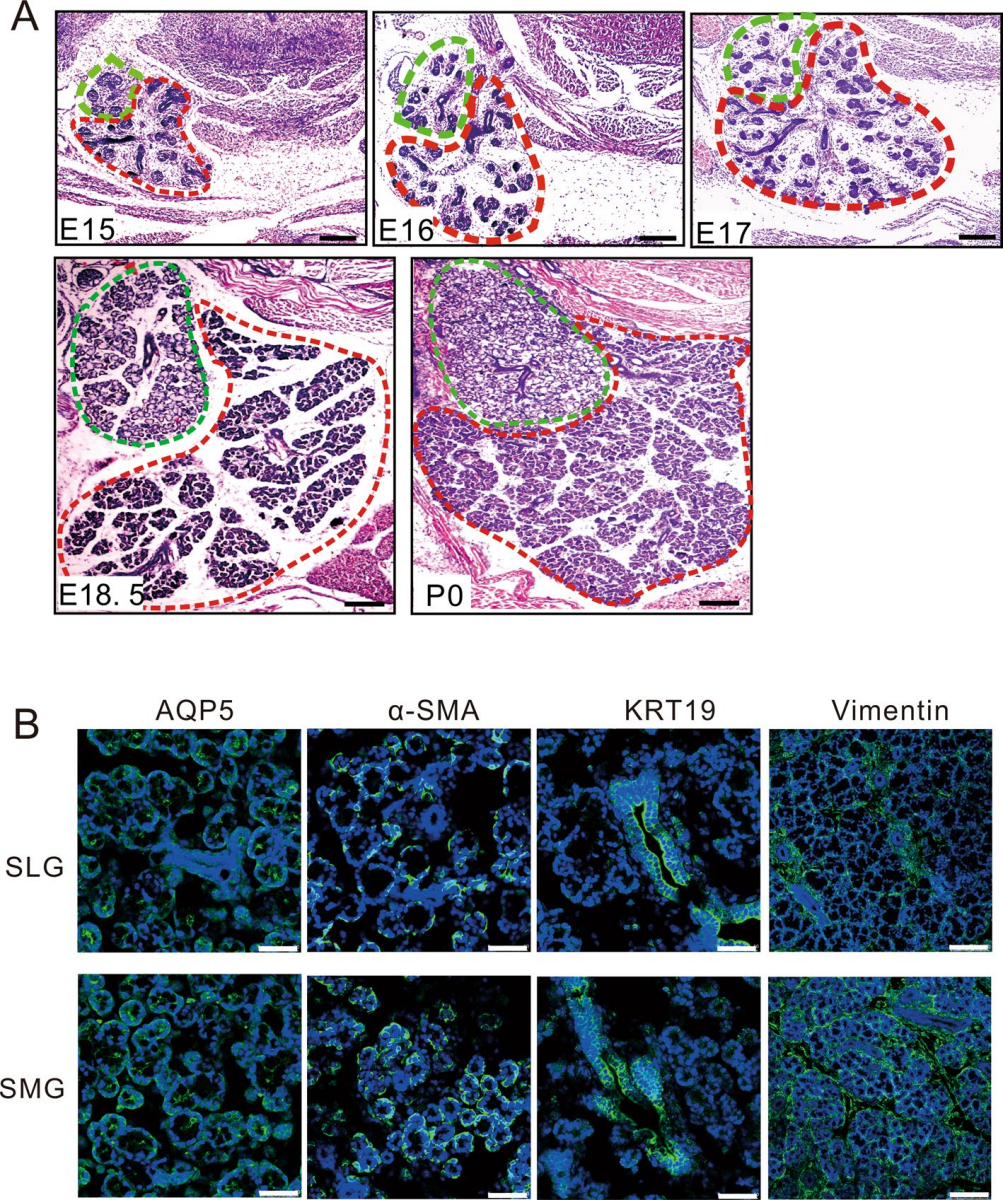
Morphology and marker expression in SMG and SLG. **A** H&E staining of a mouse embryonic submandibular gland (SMG, red dotted line) and sublingual gland (SLG, green dotted line) in the middle-late phase of development. Remarkably differentiated acinar cells are shown on E18.5, characterized by SMG with a majority of serous acinar cells and SLG with a majority of mucous acinar cells. **B** Salivary gland markers were detected by fluorescent staining. Aquaporin (AQP) 5 protein was localized in the cytomembrane of acinar cells. Smooth muscle α-actin (α-SMA) was revealed in the cytoplasm of myoepithelial cells and basal duct cells. Cytokeratin (KRT)19 was marked in luminal duct cells cytoplasm. Mesenchymal marker vimentin was expressed in mesenchymal cells and a few basal epithelial cells cytoplasm. Bar, 200 μm

The initial bud of mouse SMG and SLG undergoes extensive branching and cytodifferentiation, eventually leading to the formation of the duct lumen on E15 and mature acini on E18. Hematoxylin–eosin (HE) staining showed that the morphology of the SLG acini was dominated by serous acinar cells, while that of the SLG acini was dominated by mucous acinar cells at E17.5 [32]. On histological staining, we could detect the classic significant differences between serous acinar cells, characterized by round nuclei that are oriented toward the center part of the cells, and mucous acinar cells characterized by flattened nuclei located basally in the cells on E18.5 (Fig. 1A). Immunofluorescence staining revealed that these antibodies enabled a distinction to be made between the structural components of SMG and SLG on P0 (Fig. 1B). The acinar cell differentiation marker aquaporin (AQP) 5 protein was monitored in the acinar cell membrane. Cytokeratin (KRT)19, which initially marked the ductal lineage at E12, was located in the cytoplasm of luminal duct cells. Smooth muscle α-actin (α-SMA) was detected in the cytoplasm of myoepithelial and basal duct cells, contributing to the contractile force to induce saliva secretion out of the acini. During SMG and SLG development, the function of the mesenchyme received less attention. We found that the mesenchymal marker vimentin was expressed in the cytoplasm of mesenchymal cells and a few basal epithelial cells.

### Mouse iSMGCs or iSLGCs immortalized using the retroviral vector SSR#41 can be maintained in long-term culture

**Fig. 2:**
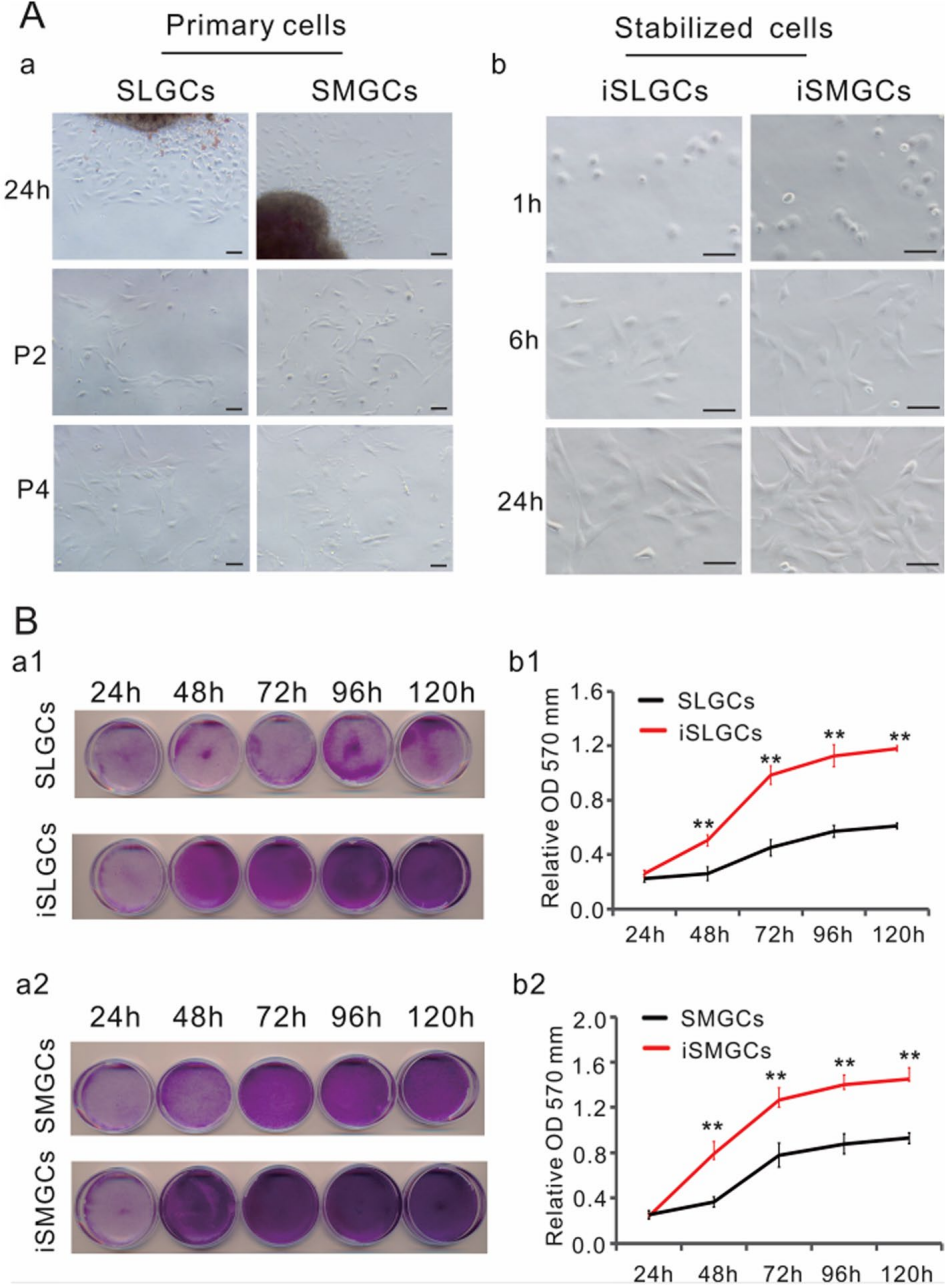
Isolation and immortalization of SMGCs (iSMGCs) and SLGCs (iSLGCs). **A** Primary SMGCs or SLGCs were isolated on P0 SMG or SLG (captured under transmission light) and cultured in complete DMEM medium. **a** Morphology of primary cells at 24 h after planting, in addition to passage 3 (p2) and p4. **b** Establishment iSMGCs and iSLGCs. Primary SMGCs or SLGCs were infected with retrovirus SSR#41 and selected in hygromycin-containing medium for 3 days. The surviving cells were designated as iSMGCs or iSLGCs. The immortalized cells of iSMGCs or iSLGCs were photographed at the indicated time and maintained indefinitely (passage 25 is shown). **B** Cell proliferation was assessed via crystal violet staining assay. The same number of primary SMGCs or SLGCs (passage 2) and iSLGCs (a1) or iSMGCs (a2) were seeded at a low density and fixed with paraformaldehyde for crystal violet staining at the indicated timepoints. The stained cells of iSMGCs (b1) and iSLGCs (b2) were dissolved in 10% acetic acid and detached for OD measurement to quantitatively determine at A_570 nm_ to A_590 nm_. The assays were performed in triplicate. **P < 0.01

For decades, research has mainly focused on branching morphogenesis during SMG and SLG development. To further study the molecular cell biology of salivary gland functions, we isolated primary SMGCs or SLGCs and then established immortalized cell lines. A certain number of cells dissociated from the tissue mass at 24 h and grew confluent at 96 h without enzyme digestion (Fig. 2A-a). Unfortunately, primary SMGCs and SLGCs could not grow well for more than four passages. It is well known that the SSR#41 vector, which expresses the SV40 T antigen, is an efficient clone carrier to establish reversibly immortalized cell lines with FLP recombinase [24]. The surviving cells selected by hygromycin B were designated as iSMGCs or iSLGCs and replicated well separately (Fig. 2A-b). We next examined differences in the proliferation ability of primary cells (passage 1) and immortalized cells (passage 25) using a crystal violet staining assay. A similar initial cell density was used, but the immortalized cells reached confluence faster than the primary cells (Fig. 2B-a). Acetic acid dissolved the stained cells, and then quantitative assessment revealed that the immortalized cells had a significantly higher cell number at each timepoint than did primary cells (Fig. 2B-b). These results showed that immortalized iSMGCs or iSLGCs were successfully established and that they could be used for further research.

### iSMGCs and iSLGCs express salivary gland markers

**Fig. 3:**
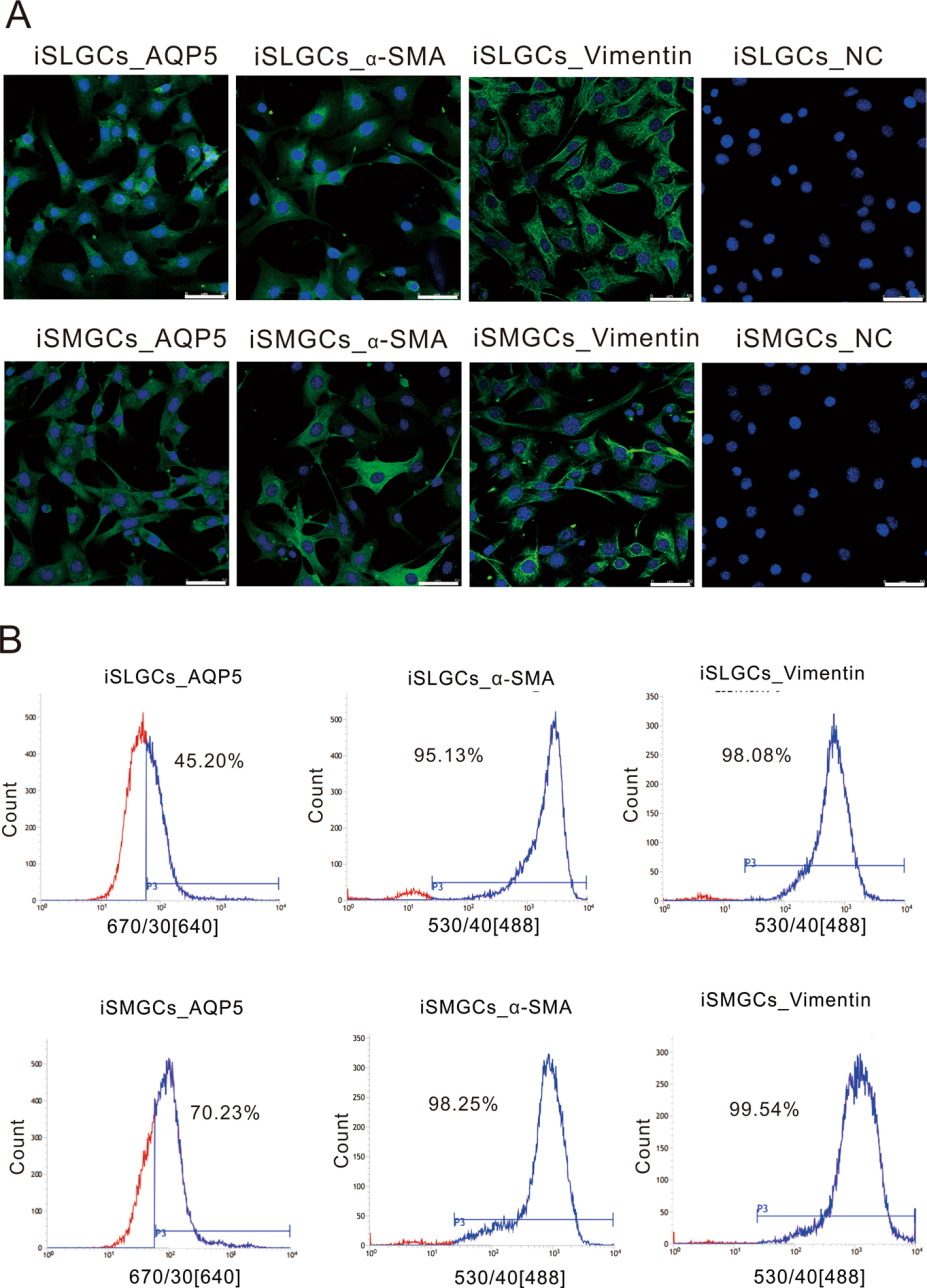
Characterization of iSMGCs and iSLGCs. **A** Expression of salivary gland markers in iSMGCs and iSLGCs. Cells were fixed with paraformaldehyde and incubated with primary and fluorescently labeled secondary antibodies in sequence. Stains without primary antibodies were used as the negative control. Representative results were photographed by laser scanning confocal microscope. Bar, 50 μm. **B** Flow cytometry analysis of the expression of salivary gland markers revealed the percentage of specific markers in iSMGCs and iSLGCs

Immunofluorescence staining showed that the expression of Krt19, α-sma, and Aqp5 could be detected in the cytomembrane and cytoplasm of iSMGCs and iSLGCs (Fig. 3A). To further investigate the proportion of these markers in iSMGCs and iSLGCs, cells were labeled directly using fluorescent antibodies or indirectly using flow cytometry (FCM) analysis. As shown in Fig. 3B, both the iSMGCs and the iSLGCs were positive for these salivary gland markers. The percentages of cells positive for specific markers were as follows: 98.25% α-sma, 70.23% Aqp5, and 99.54% vimentin in iSMGCs, and 95.13% α-sma, 45.20% Aqp5, and 98.08% vimentin in iSLGCs. However, Krt7- and Krt19-positive cells were barely detected (Additional file 1: Fig. S1). Collectively, the above results confirm that iSMGCs and iSLGCs partially presented characteristics of salivary glands.

### FLP recombinase-mediated removal of SV40 T antigen decreases the proliferative activity of iSMGCs and iSLGCs

**Fig. 4:**
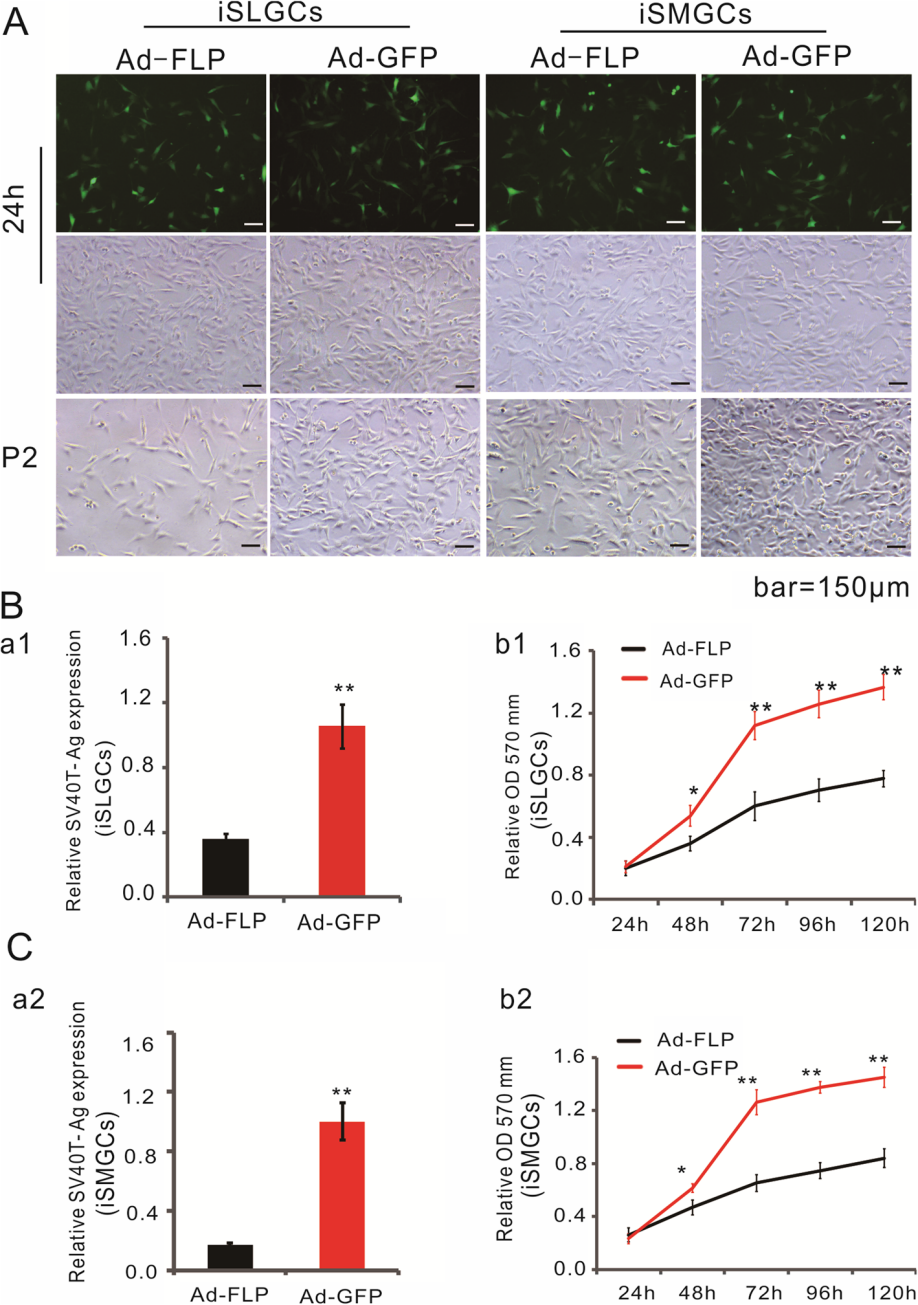
FLP recombinase-mediated removal of SV40 T antigen decreases proliferative activity of iSMGCs and iSLGCs. **A** FLP recombinase-mediated removal of SV40 T antigen in iSMGCs or iSLGCs. Cells were infected with Ad-FLP or Ad-GFP as the control. Quantity and intensity of green fluorescent cells was high at 24 h during these treatments. The growth status of Ad-FLP-transduced cells or Ad-GFP-treated cells is shown at passage 2 (p2). **B** SV40 T-antigen removal efficiency and the effects of multiplication capacity in cells. RT-qPCR confirmed that Ad-FLP decreased SV40 T-antigen expression significantly compared with Ad-GFP treatment. Meanwhile, the multiplication capacity of cells declined and was accompanied by the removal of the SV40 T antigen. Each assay condition was analyzed using a minimum of three independent experiments. *P < 0.05, **P < 0.01

Cell immortalization via SV40 large T antigen in the retroviral vector SSR#41 could be effectively reversed by the action of FLP recombinase. To obtain reversely immortalized cell lines, we constructed a recombinant adenoviral vector (Ad-FLP) and used it to transfect iSMGCs or iSLGCs. We observed a parallel high infection efficiency in cells infected with Ad-FLP or Ad-GFP (Fig. 4A). RT-PCR analysis confirmed that we efficiently removed the SV40 T antigen from Ad-FLP-infected iSMGCs or iSLGCs, but not from Ad-FLP-infected cells (Fig. 4B-a1, a2). To test whether the growth status of AdFLP-infected iSMGCs or iSLGCs would be affected, cell proliferation was assessed using CCK8 quantitative analysis. We found that the proliferative capacity of both AdFLP-infected iSMGCs and iSLGCs was significantly reduced compared with that of AdGFP-transduced iSMGCs or iSLGCs, separately (*P* < 0.001) (Fig. 4B-b1, b2). Tumorigenicity assays were carried out in nude mice to exclude the risk of tumorigenesis (data not shown). Taken together, these results indicate that two immortalized cell lines were established, which could be reversed by FLP recombinase, but their proliferative activity was impaired.

### RNA-seq revealed differential gene expression between iSMGCs and iSLGCs

**Fig. 5:**
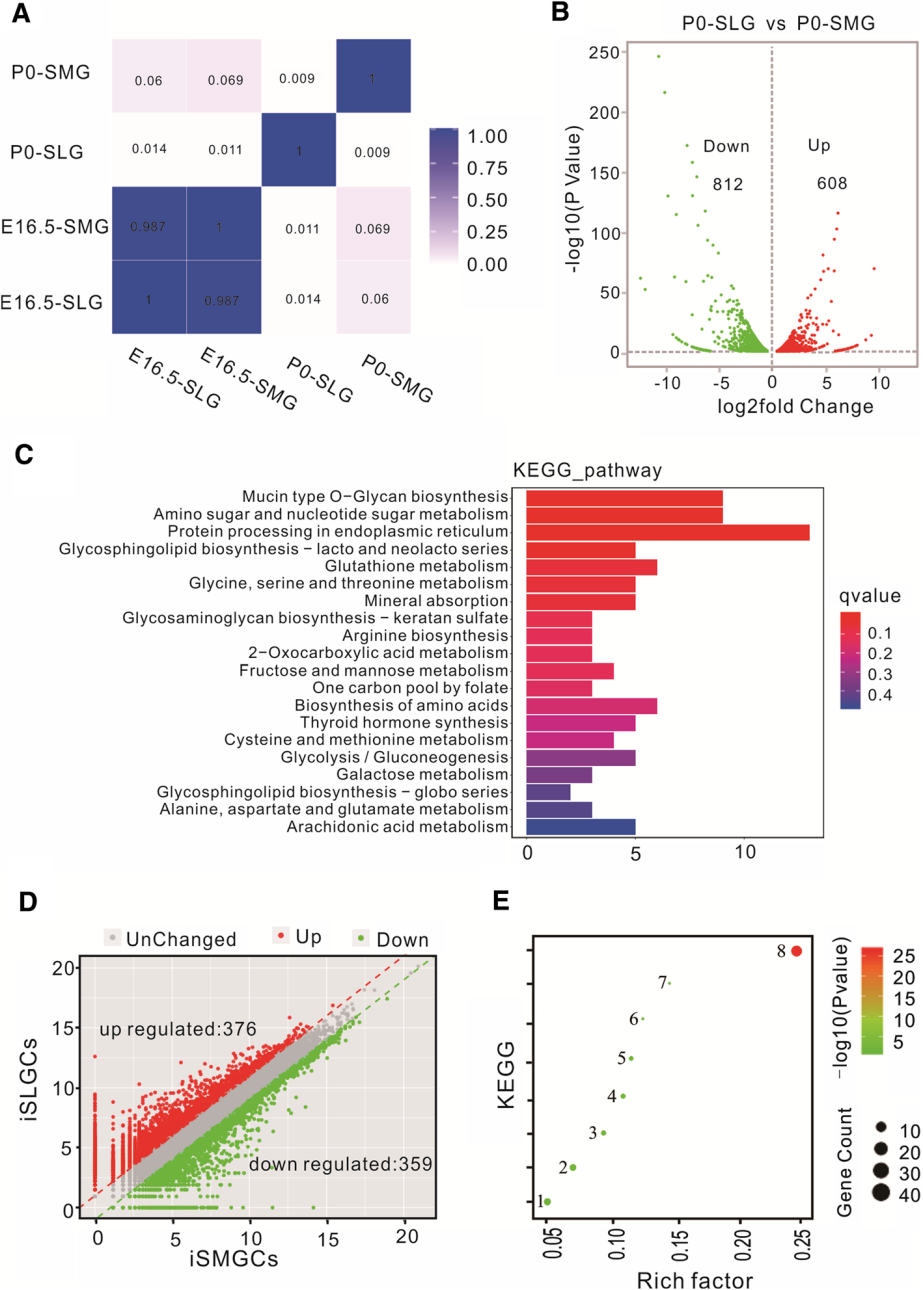
Differentially expressed gene analysis (DEG) and KEGG enrichment by RNA-seq. **A** Correlation heatmap showing correlation between SLG and SMG on E16.5 and P0. **B** In total, 608 upregulated (red dots) and 812 downregulated (green dots) DEGs with *P* < 0.001 were counted between SLG and SMG. **C** The top 20 KEGG pathways between SLG and SMG on P0 are listed. **D** RNA-seq analysis revealed upregulated (376) and downregulated (359) DEGs in iSMGCs, compared with in iSLGCs. **E** KEGG enrichment included eight signaling pathways based on the DEGs between iSMGCs and iSLGCs: 1–8 represent cytokine–cytokine receptor interaction, Jak–STAT signaling pathway, leishmaniasis, legionellosis, cysteine and methionine metabolism, hippo signaling pathway, proximal tube bicarbonate reclamation, and ribosome, respectively

To conduct global analysis between the organs and corresponding cell lines, RNA-seq of the E16.5 and P0 salivary gland was performed. RNA-seq analysis exhibited that the correlation between SMG and SLG was *r*^2^ = 0.987 on E16.5 and *r*^2^ = 0.009 on P0 (Fig. 5A). These data suggest that there were over 1000 DEGs between the SMG and SLG on P0 (Fig. 5B). To evaluate these differences, a volcano plot was constructed showing the significantly DEGs with more than twofold change in their expression levels and *P*-value < 0.001. Compared with the SLG, a total of 812 downregulated and 608 upregulated genes were identified in the SMG. KEGG pathway enrichment of the top 20 DEGs was performed (Fig. 5C). KEGG pathways were specifically enriched in metabolism, including amino sugar and nucleotide sugar metabolism, glutathione metabolism, glycine, serine and threonine metabolism, and cysteine and methionine metabolism. These data confirm that the SMG and SLG are two important metabolic organs. The SMG and SLG exhibited some similar gland marker protein location and remarkable metabolic pathway DEG enrichment on KEGG analysis on P0.

To systematically assess the differences in gene expression between iSMGCs and iSLGCs, differentially expressed genes (DEGs) were analyzed. First, the analysis of transcriptomic data revealed 735 DEGs between iSMGCs and iSLGCs involved in cytokine–cytokine receptor interaction, the Jak–STAT signaling pathway, leishmaniasis, legionellosis, cysteine and methionine metabolism, the Hippo signaling pathway, proximal tubule bicarbonate reclamation, and ribosome (Fig. 5D and E). The KEGG enrichment analysis revealed that the ribosome was the most enriched. These data indicate the usefulness of iSMGCs and iSLGCs in future metabolism-related research.

### DEG analysis and verification upon BMP9/Gdf2 stimulation

Radiation- or duct-calculus-induced salivary gland fibrosis leads to xerostomia. Previous studies have suggested that TGF-β contributes to glandular fibrosis [22]. Recent studies identified a novel functional role for BMP9 in the treatment of cardiac or liver fibrosis. In our study, we also found weakly positive expression of BMP9 in duct and mesenchyme mature SMG and SLG (Additional file 1: Fig. S2). Our previous results also showed the expression of *Bmp9* was upregulated by duct-calculus-induced salivary gland fibrosis (Additional file 1: Fig. S3). To further evaluate whether exogenous BMP9 supplementation could play a role in the fibrotic salivary gland, an adenovirus expressing BMP9 (Ad-BMP9) was transfected in iSMGCs or iSLGCs for 24 h. Cell RNA samples were used to conduct RNA sequencing (RNA-seq) experiments.

**Fig. 6:**
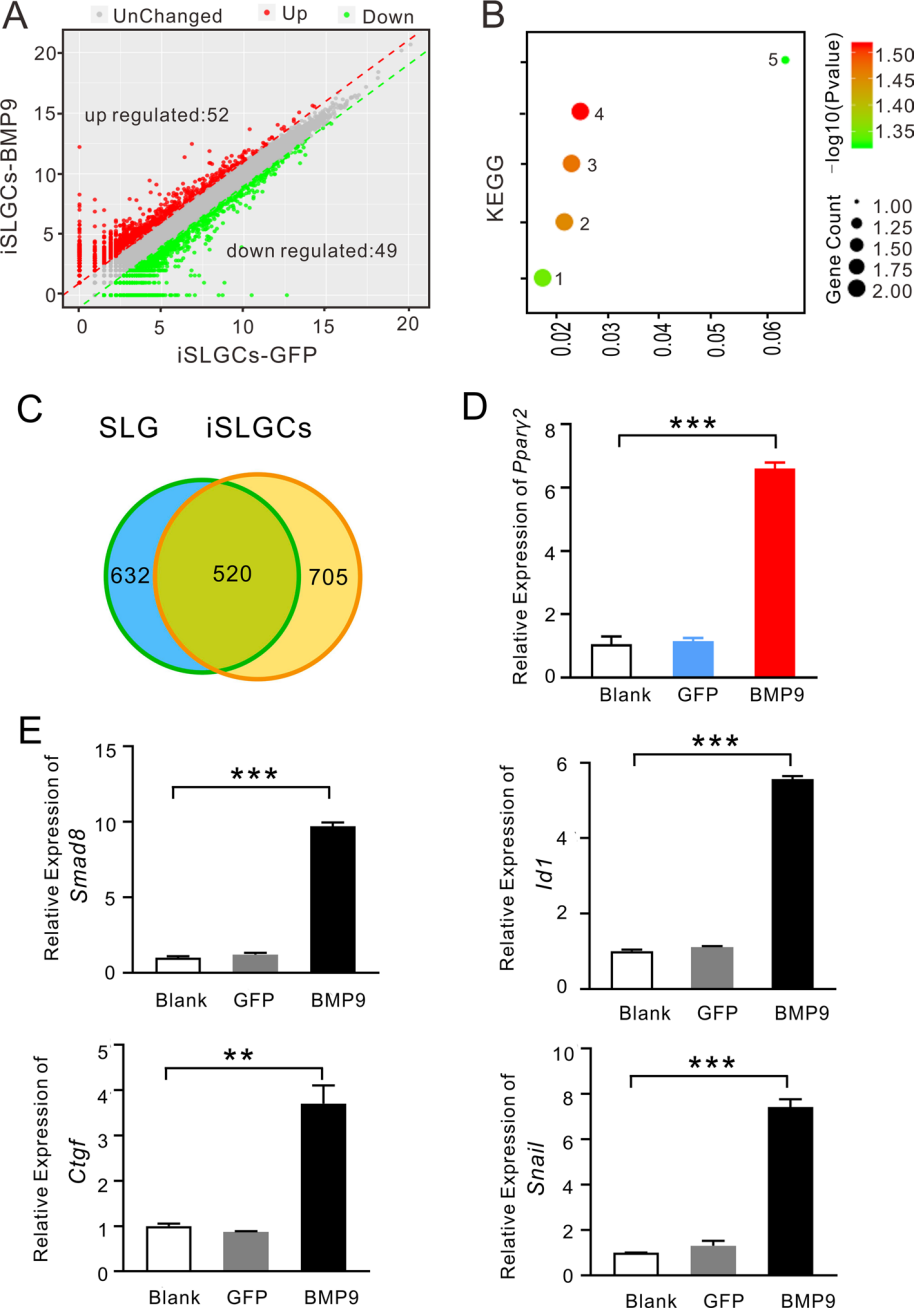
Differentially expressed gene analysis (DEG) and indicated gene verification in SLGCs. **A** The significantly upregulated (52) and downregulated (49) DEGs induced by Ad-BMP9 in iSLGCs. **B** KEGG enrichment signaling pathways included: amoebiasis (1), small-cell lung cancer (2), protein digestion and absorption (3), PPAR signaling pathway (4), and thiamine metabolism (5). **C** Overlap of metabolic gene expression between the cell line and tissues. There were 520 metabolism-related genes coexpressed between SLG and iSLGCs. **D** The expression of *Pparγ-2* was increased upon the induction of Ad-BMP9. **E** The upregulated expression levels of *Id1 *and *Smad8* were confirmed using RT-qPCR demonstrating the activity of BMP9. BMP9 stimulated *Ctgf *and *Snai1* expression in iSLGCs. All experiments were repeated at least three times. **P < 0.01, ***P < 0.001

**Fig. 7:**
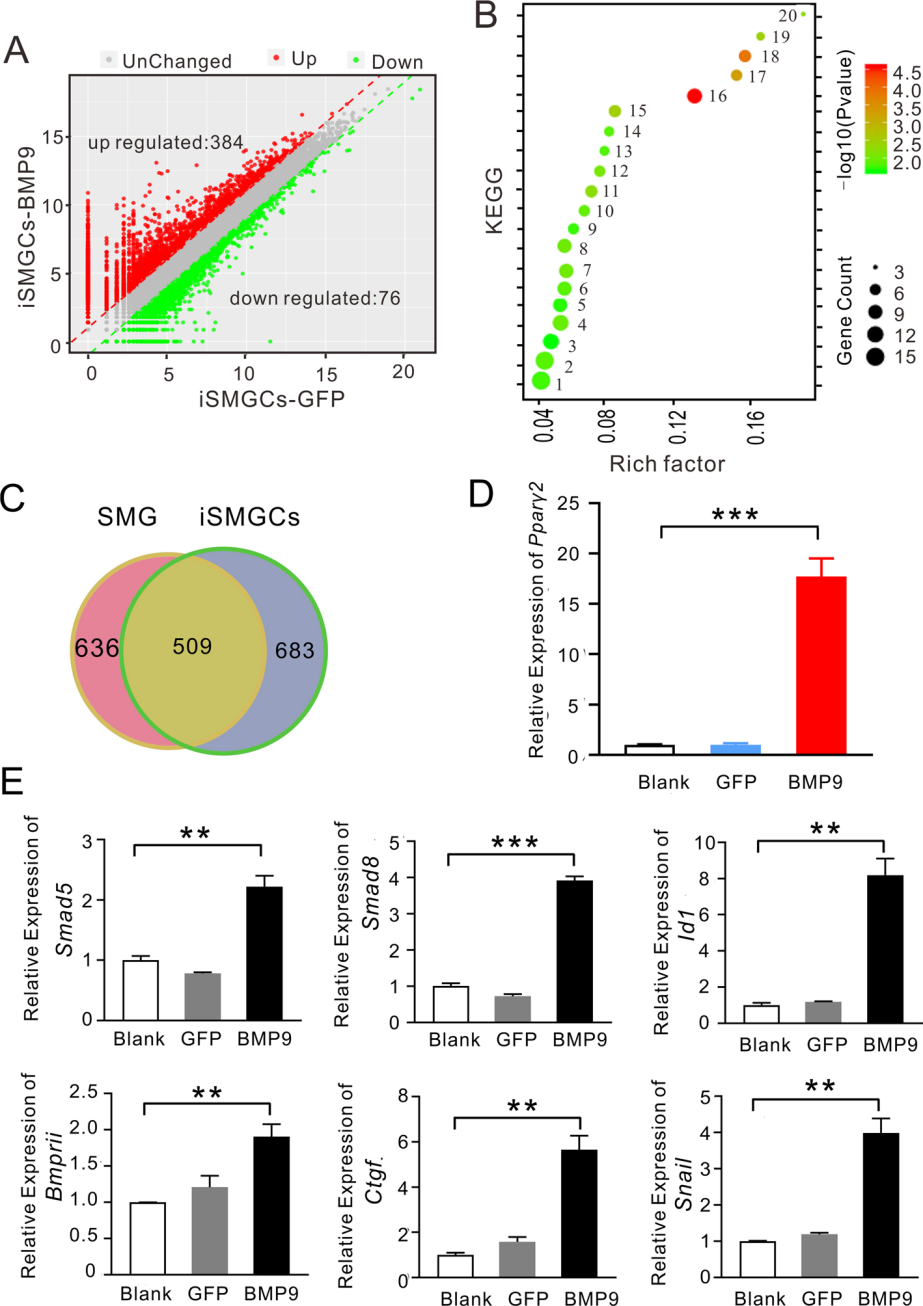
Differentially expressed gene (DEG) analysis and indicated gene verification in SMGCs. **A** The significantly upregulated (384) and downregulated (76) DEGs induced by Ad-BMP9 in iSMGCs. The significantly DEG (red and green dots) with two or more fold changes in expression and *P* < 0.05. **B** KEGG enrichment signaling pathway, where 1–20 represent: PI-3K-Akt signaling pathway, human papillomavirus infection, ras signaling pathway, Rap1 signaling pathway, axon guidance, cell adhesion molecular (CAMs), hippo signaling pathway, gastric cancer, retinol metabolism, rheumatoid arthritis, hematopoietic cell line, melanoma, synaptic vesicle cycle, glutathione metabolism, ECM–receptor interaction, PPAR signaling pathway, mineral absorption, fatty acid degradation, collection duct acid secretion, and glycosphingolipid biosynthesis-globo and isoglobo series. **C** 509 metabolism-related genes coexpressed between SMG and iSMGCs. **D** The expression of *Pparγ-2* was increased upon Ad-BMP9 induction. **E** The upregulated expression levels of *Id1*, *Smad5*, and *Smad8* were confirmed using RT-qPCR, demonstrating the activity of BMP9. BMP9 stimulated Ctgf and Snai1 expression in iSLGCs. All experiments were repeated at least three times. **P < 0.01, ***P < 0.001

First, we identified 101 significant DEGs with an expression change of more than twofold in iSLGCs (Fig. 6A) and 460 significant DEGs in iSMGCs after Ad-BMP9 treatment (Fig. 7A). KEGG pathway analysis suggested that the DEGs in iSLGCs were primarily enriched in protein digestion and absorption, thiamine metabolism, and the PPAR signaling pathway (Fig. 6B). Analysis of the DEGs in SMGCs after Ad-BMP9 treatment showed that metabolism-related pathways were enriched, including retinol metabolism, glutathione metabolism, fatty acid degradation, and mineral absorption (Fig. 7B). PPAR-γ is a key transcription factor of PPARs (α, β/δ, and γ), which is closely related to intracellular antiinflammatory effects, metabolic homeostasis, and fibrosis [33]. Interestingly, a common PPAR signaling pathway was found between iSMGCs versus BMP9-treated iSMGCs and iSLGCs versus BMP9-treated iSLGCs on KEGG enrichment analysis (Figs. 6B and 7B).

Then, we examined the metabolism-related genes in the tissue and cell lines. We found that 520 metabolism-related genes were coexpressed in the SLG (632 metabolism-related genes) and iSLGCs (705 metabolism-related genes), while 509 metabolism-related genes were coexpressed in the SMG (636 metabolism-related genes) and iSMGCs (683 metabolism-related genes) (Figs. 6C and 7C). Cysteine and methionine metabolism was the common KEGG pathway between these two cell lines compared with SMG versus SLG.

PPARγ-2 plays an important role in preadipose glucose metabolism and lipid metabolism. The increased expression of PPARγ-2 induced by BMP9 in iSLGCs or iSMGCs contributes to a better understanding of the mechanisms of lipid metabolism in the salivary gland (Figs. 6D and 7D). Next, the increased expression levels of *Id1*, *Smad1/5/8*, and *BmprII *were confirmed using RT-qPCR demonstrating the activity of BMP9 (Fig . 6E and 7E). We found that the expression levels of both* Id1* and *Smad 8* were significantly upregulated after Ad-BMP9 transfection in both iSLGCs and iSMGCs, while those of *Smad5* and *BmprII *were only significantly upregulated in BMP9-stimulated iSMGCs. Connective tissue growth factor (Ctgf) is a secreted glycoprotein produced by fibroblasts, myofibroblasts, and endothelial cells. Through various regulatory modulatory interactions, Ctgf regulates cellular fibrosis stimulated by TGF-β [34, 35]. In our study, BMP9 stimulated* Ctgf *expression in both iSLGCs and iSMGCs. Snail (*Snai1*) responds to TGF-β-induced epithelial–mesenchymal transition (EMT) by repressing E-cadherin expression, and is expressed in mesenchymal stromal fibroblasts but not in normal epithelial cells [36, 37]. In the SMG duct ligation model, the significantly increased expression of *Snail *was accordant with fibrotic progression. After transfection with Ad-BMP9 in iSLGCs or iSMGCs, BMP9 consistently upregulated *Snail* expression (Figs. 6E and 7E). Overall, these results partially demonstrated the metabolic and fibrotic effects of BMP9 on the salivary gland resulting from modulation of multiple genes.

## Discussion

Although it is widely known that duct-calculus-induced salivary gland fibrosis leads to xerostomia and metabolic disorder, the lack of a relevant salivary gland cell models hampers our understanding of the biology and therapy of salivary gland fibrosis. To overcome the limited lifespan of the primary SMGCs or SLGCs, here we successfully immortalized iSMGCs and iSLGCs using the SSR#41 vector, expressing the SV40 T antigen flanked with FRT sites, and investigated the metabolic and fibrotic functions of the salivary gland upon BMP9 stimulation.

The salivary gland is a favorable model to verify branching development in vitro and in vivo. In the past decades, a large number of detailed mechanisms of salivary gland morphogenesis have been recognized, including fibroblast growth factor (FGF) signaling, epidermal growth factor (EGFR) signaling, and Hippo signaling [8, 38, 39].

However, salivary gland dysfunction (i.e., xerostomia) is a common disease that leads to oral infection and poor digestion, lacking in-depth study. Until now, treatments with sialogogues and saliva substitutes are limited, as they partly relieve dry-mouth symptoms but do not restore glandular function. Cultured acinar cells or acinar precursor cells were the primary cells used to explore the mechanisms of damaged acinar in the past several decades. However, acinar cells, as terminally differentiated cells, could not grow well in vitro, and committed differentiation of acinar precursor cells was also a challenge. Fortunately, more and more research has confirmed that residual surviving acinar cells, ductal stem cells, and even mesenchymal cells could be investigated as the progenitor cells responsible for acinar cell regeneration. Thus, we sought to a establish stable cell lines combining good growth and relatively mature differentiated phenotype. Interestingly, mucous and serous acinar cells were detected from E17.5, close to mouse birth, as demonstrated by histological staining, presenting the end of differentiation or “functional acquisition.” RNA-seq analysis demonstrated that there were more than 1000 DEGs and extremely low correlation between the SMG and SLG on P0. Moreover, immunofluorescent staining verified structural components of differentiation markers on P0. These data suggest that SMG and SLG reached the end of differentiation on P0. Compared with developing fetuses, isolation primary cells from newborn pups may be more ethical. Owing to the above reasons, we selected the phase of P0 to establish stable cell lines.

We previously found that the use of SV40 T antigen transduced into primary cells to establish reversibly immortalized cell lines from various tissues is generally nontumorigenic. Otherwise, SV40 T-immortalized vector with flanked with FRT sites could abolish the SV40 T antigen efficiently reversed by Ad-FLP transfection. The immortalized cell pool iSMGCs and iSLGCs did not show tumorigenic risk. We also obtained reversely immortalized cell lines transfecting Ad-FLP to iSMGCs or iSLGCs. CCK8 quantitative analysis revealed that FLP-mediated removal of SV40 T antigen significantly impaired the proliferative activity of iSMGCs or iSLGCs. Several markers are identified in different cell components of salivary gland in the epithelium (ducts, acini) and mesenchyme [40, 41]. We detected some salivary gland markers on P0, and AQP5, KRT19, α-SMA, and Vimentin were also expressed in iSMGCs or iSLGCs. FCM analysis further confirmed the ratio of these markers. As expected, a large proportion of cells originating from the mesenchyme expressed Vimentin and α-SMA. To rule out the effects of the isolation method, we reviewed the types of primary SMG or SLG cells isolated using enzyme digestion and analyzed them using single-cell sequencing [42–44]. These data showed that the majority of cells were mesenchymal cells. Surprisingly, the positive proportion of AQP5 approached 50% both in iSMGCs and iSLGCs, inconsistent with the extremely high expression of mesenchymal markers. Although vimentin is scattered in the surrounding cells of ducts and acini, it cannot be neglected that these cells might have a stem cell function owing to positive expression in basal cells. Thus, we believe iSMGCs and iSLGCs reproduce SMG and SLG cell function, respectively.

Both SMG and SLG have ductal structures that open into the oral cavity, producing saliva to lubricate the mouth and aid in food digestion [45]. The parotid gland contributes as much as 60% of the stimulated flow by tasting or chewing with rich amylase and proline-rich proteins (PRPs) [3, 46]. The protein contents identified in parotid could not be deduced in submandibular or sublingual secretion owing to the different developmental origin. Some universal proteins have been reported in the three major salivary glands; mucins (encoded by Muc5b and Muc7) occur only in SMG and SLG [3]. SMG and SLG mainly contribute to the resting salivary flow, and the excretion of saliva is of a relatively stable amount and composition. The secretion of proteins identified in SMG or SLG is poorly understood. In humans, ductal anatomy prevents the proteomic analysis of pure submandibular or sublingual secretion. In mice, the amount of submandibular secretion collected though the caruncula salivaris is insufficient for proteomic analysis. Therefore, research of SMG and SLG metabolic functions is challenging. Currently, few studies have assessed SMG and SLG metabolic functions through cell line analysis in vitro. In this work, we performed global analysis of the SMG and SLG using transcriptomic data. We found that an abundance of metabolism-related genes was expressed in these glands at P0. Furthermore, KEGG pathway enrichment analysis of the top 20 DEGs revealed metabolism-related genes, including those relating to amino sugar and nucleotide sugar metabolism, glutathione metabolism, glycine, serine, and threonine metabolism, and cysteine and methionine metabolism. Transcriptomic data analysis identified 735 DEGs between iSMGCs and iSLGCs, and KEGG analysis showed that they were enriched in cysteine and methionine metabolism. Lactate dehydrogenase (LDH) plays a crucial role in anaerobic glycolysis, which is predominant in tumor cells [47]. The level of intracellular LDH activity in iSMGCs and iSLGCs requires further study. Here we demonstrated that the iSMGCs and iSLGCs effectively reproduced the SMG and SLG metabolic functions partly, although further investigation is warranted to determine which pathways can be attributed to SMG and which to SLG.

Treatment of salivary gland fibrosis, which lacks a clear pathogenic mechanism, is a common clinical challenge. It has recently attracted significant attention concerning the use of gene delivery to rescued salivary function [13, 48]. BMP9 plays a role in various organ fibroses, including liver fibrosis, cardiac functions through classical BMPs/Smads signaling pathways and nonclassical pathways, and a variety of signals and cytokines [49]. BMP9 also plays important roles in inhibiting hepatic glucose production, inducing the expression of key enzymes of lipid metabolism. Here we first established salivary gland immortalized cell lines to explore whether exogenous supplementation of BMP9 affects metabolism and fibrosis. The transcriptome sequencing data revealed enrichment in protein digestion and absorption, thiamine metabolism in BMP9-stimulated iSLGCs and retinol metabolism, glutathione metabolism, fatty acid degradation, and mineral absorption in BMP9-stimulated iSMGCs. Interestingly, BMP9 could activate the PPAR signaling pathway in neither iSMGCs nor iSLGCs, leading to increased *Pparγ-2 *expression but not *PPAR-α* or *PPAR-β *expression. These findings indicate that BMP9 might provide new insight into salivary gland metabolism. Furthermore, our results demonstrate that BMP9 activated fibrosis by upregulating *Ctgf* and *Snail*. Moreover, we detected the BMP9-specific receptor tentatively in iSMGCs and iSLGCs. ActrII, *BmprIa*, and* BmprII* were expressed, while *AcrrIIb*, *BmprIb*, *Alk1*, and *Alk2* were detected at extremely low levels (data not shown). Phosphorylation of SMAD1/5/8 promotes SMAD4 translocation to the nucleus, followed by the activation of SMAD-response elements [50]. A relatively low mRNA level of *Smad8* was detected in iSMGCs and iSLGCs, which increased following BMP9 stimulation. The feedback inhibitor *Smads*, *Smad6* and *Smad7*, also presented higher expression in BMP9-stimulated iSMGCs and iSLGCs (data not shown). To some extent, our data show a tentative exploration of the fibrotic function of BMP9 in the salivary gland, and the corresponding signals. Whether or not BMP9 ablation regulates salivary gland function warrants thorough future investigation.

Inactivation of tumor suppressor genes due to promoter methylation plays an important role in salivary gland adenoid cystic carcinoma [51, 52]. Since exogenous conduction of SV40 T antigen in iSMGCs and iSLGCs may increase the risk of tumorigenesis, we confirmed the absence of tumor-like masses in vivo. Therefore, more evidence is required to determine whether iSMGCs and iSLGCs are suitable for the study of salivary gland carcinoma.

## Conclusion

In summary, we established reversely immortalized mouse salivary gland cell lines, named iSMGCs and iSLGCs, and performed a global transcriptomic analysis of these cell lines. Moreover, exogenous administration of BMP9 activated metabolism and fibrosis in iSMGCs and iSLGCs. Our findings provide a foundation for metabolism- and fibrosis-related research of the salivary gland.

## Supplementary Information

**Additional file 1: Figure S1.** Flow cytometry analysis of the bare expression of Krt7 and Krt19 in iSMGCs or SLGCs. **Figure S2.** Immunofluorescence staining exhibited positive expression of BMP9 in ducts and mesenchyme in SMG and SLG at four and eight weeks postnatal. Bar = 75 μm. **Figure S3.** Upregulation of* Bmp9 *and *Col1a1* in response to submandibular gland ductal ligation. (A) Gross morphology of submandibular gland after a seven day duct ligation. (B) histomorphology of SMG after a seven day duct ligation were examined by H&E staining. (C) The mRNA expression of *Bmp9* and *Col1a1 *were increased after a seven day duct ligation and detected by RT-qPCR. All experiments were repeated at least three times and compared with a control. **P < 0.01, ***P < 0.001.

## Data Availability

All data generated or analyzed during this study are included in this published article and its additional information files.

## Abbreviations

SMG: Submandibular gland
SLG: Sublingual gland
SMGCs: Mouse primary SMG cells
SLGCs: Mouse primary SLG cells
iSMGCs: Immortalized mouse primary SMG cells
iSLGCs: Immortalized mouse primary SLG cells
Ad-BMP9: Adenoviral construct encoding bone morphogenetic protein 9
Ad-GFP: Adenoviral construct encoding green fluorescent protein
Gdf2: Growth differentiation factor 2
Ctgf: Connective tissue growth factor
Snail1: Snail family transcriptional repressor 1
Fgf: Fibroblast growth factor
Egfr: Epidermal growth factor receptor
Aqp5: Aquaporin 5
Krt19: Keratin 19
Krt7: Keratin 7
α-sma: Smooth muscle α-actin
Tgf-β: Transforming growth factor-β
PPARs: Peroxisome proliferator-activated receptors
BmprII: Bone morphogenetic protein receptor type 2
Id-1: Inhibitor of DNA binding 1

## References

[1] Denny PC, Ball WD, Redman RS (1997). Salivary glands: a paradigm for diversity of gland development. Crit Rev Oral Biol Med.

[2] Knosp WM, Knox SM, Hoffman MP (2012). Salivary gland organogenesis. Wiley Interdiscip Rev Dev Biol.

[3] Carpenter GH (2013). The secretion, components, and properties of saliva. Annu Rev Food Sci Technol.

[4] Patel VN, Hoffman MP (2014). Salivary gland development: a template for regeneration. Semin Cell Dev Biol.

[5] Holmberg KV, Hoffman MP (2014). Anatomy, biogenesis and regeneration of salivary glands. Monogr Oral Sci.

[6] Lombaert IMA, Hoffman MP (2010). Epithelial stem/progenitor cells in the embryonic mouse submandibular gland. Front Oral Biol.

[7] Häärä O, Koivisto T, Miettinen PJ (2009). EGF-receptor regulates salivary gland branching morphogenesis by supporting proliferation and maturation of epithelial cells and survival of mesenchymal cells. Differentiation.

[8] Tsau C, Ito M, Gromova A, Hoffman MP, Meech R, Makarenkova HP (2011). Barx2 and Fgf10 regulate ocular glands branching morphogenesis by controlling extracellular matrix remodeling. Development (Cambridge, England).

[9] Matsumoto S, Kurimoto T, Taketo MM, Fujii S, Kikuchi A (2016). The WNT/MYB pathway suppresses KIT expression to control the timing of salivary proacinar differentiation and duct formation. Development (Cambridge, England).

[10] Knosp WM, Knox SM, Lombaert IM, Haddox CL, Patel VN, Hoffman MP (2015). Submandibular parasympathetic gangliogenesis requires sprouty-dependent Wnt signals from epithelial progenitors. Dev Cell.

[11] Cherry CP, Glucksmann A (1959). Injury and repair following irradiation of salivary glands in male rats. Br J Radiol.

[12] Aure MH, Konieczny SF, Ovitt CE (2015). Salivary gland homeostasis is maintained through acinar cell self-duplication. Dev Cell.

[13] Hu L, Zhu Z, Hai B, Chang S, Ma L, Xu Y (2018). Intragland Shh gene delivery mitigated irradiation-induced hyposalivation in a miniature pig model. Theranostics.

[14] Mostafa S, Pakvasa M, Coalson E, Zhu A, Alverdy A, Castillo H (2019). The wonders of BMP9: from mesenchymal stem cell differentiation, angiogenesis, neurogenesis, tumorigenesis, and metabolism to regenerative medicine. Genes Dis.

[15] Zhang L, Luo Q, Shu Y, Zeng Z, Huang B, Feng Y (2019). Transcriptomic landscape regulated by the 14 types of bone morphogenetic proteins (BMPs) in lineage commitment and differentiation of mesenchymal stem cells (MSCs). Genes Dis.

[16] Song D, Zhang F, Reid RR, Ye J, Wei Q, Liao J (2017). BMP9 induces osteogenesis and adipogenesis in the immortalized human cranial suture progenitors from the patent sutures of craniosynostosis patients. J Cell Mol Med.

[17] Seeherman HJ, Berasi SP, Brown CT, Martinez RX, Juo ZS, Jelinsky S, et al. A BMP/activin A chimera is superior to native BMPs and induces bone repair in nonhuman primates when delivered in a composite matrix. Sci Transl Med. 2019;11(489). 10.1126/scitranslmed.aar4953 31019025

[18] Chen Q, Zheng L, Zhang Y, Huang X, Wang F, Li S (2021). Special AT-rich sequence-binding protein 2 (Satb2) synergizes with Bmp9 and is essential for osteo/odontogenic differentiation of mouse incisor mesenchymal stem cells. Cell Prolif.

[19] Yang Z, Li P, Shang Q, Wang Y, He J, Ge S, et al. CRISPR-mediated BMP9 ablation promotes liver steatosis via the down-regulation of PPARα expression. Sci Adv. 2020;6(48). 10.1126/sciadv.abc5022 PMC7695473 33246954

[20] Li W, Long L, Yang X, Tong Z, Southwood M, King R (2021). Circulating BMP9 protects the pulmonary endothelium during inflammation-induced lung injury in mice. Am J Respir Crit Care Med.

[21] Morine KJ, Qiao X, York S, Natov PS, Paruchuri V, Zhang Y (2018). Bone morphogenetic protein 9 reduces cardiac fibrosis and improves cardiac function in heart failure. Circulation.

[22] Woods LT, Camden JM, El-Sayed FG, Khalafalla MG, Petris MJ, Erb L (2015). Increased expression of TGF-β signaling components in a mouse model of fibrosis induced by submandibular gland duct ligation. PLoS ONE.

[23] Laoide BM, Courty Y, Gastinne I, Thibaut C, Kellermann O, Rougeon F (1996). Immortalised mouse submandibular epithelial cell lines retain polarised structural and functional properties. J Cell Sci.

[24] Luo W, Zhang L, Huang B, Zhang H, Zhang Y, Zhang F (2021). BMP9-initiated osteogenic/odontogenic differentiation of mouse tooth germ mesenchymal cells (TGMCS) requires Wnt/β-catenin signalling activity. J Cell Mol Med.

[25] Yu X, Chen L, Wu K, Yan S, Zhang R, Zhao C (2018). Establishment and functional characterization of the reversibly immortalized mouse glomerular podocytes (imPODs). Genes Dis.

[26] He TC, Zhou S, da Costa LT, Yu J, Kinzler KW, Vogelstein B (1998). A simplified system for generating recombinant adenoviruses. Proc Natl Acad Sci USA.

[27] Luo J, Deng ZL, Luo X, Tang N, Song WX, Chen J (2007). A protocol for rapid generation of recombinant adenoviruses using the AdEasy system. Nat Protoc.

[28] Taylor V, Wong M, Brandts C, Reilly L, Dean NM, Cowsert LM (2000). 5' phospholipid phosphatase SHIP-2 causes protein kinase B inactivation and cell cycle arrest in glioblastoma cells. Mol Cell Biol.

[29] Jurisic V (2020). Multiomic analysis of cytokines in immuno-oncology. Expert Rev Proteomics.

[30] Chuang YM, Dutta NK, Gordy JT, Campodónico VL, Pinn ML, Markham RB (2020). Antibiotic treatment shapes the antigenic environment during chronic TB infection, offering novel targets for therapeutic vaccination. Front Immunol.

[31] Svensson MN, Andersson KM, Wasén C, Erlandsson MC, Nurkkala-Karlsson M, Jonsson IM (2015). Murine germinal center B cells require functional Fms-like tyrosine kinase 3 signaling for IgG1 class-switch recombination. Proc Natl Acad Sci USA.

[32] Metwalli KA, Do MA, Nguyen K, Mallick S, Kin K, Farokhnia N (2018). Interferon regulatory factor 6 is necessary for salivary glands and pancreas development. J Dent Res.

[33] Lefere S, Puengel T, Hundertmark J, Penners C, Frank AK, Guillot A (2020). Differential effects of selective- and pan-PPAR agonists on experimental steatohepatitis and hepatic macrophages(☆). J Hepatol.

[34] Richeldi L, Fernández Pérez ER, Costabel U, Albera C, Lederer DJ, Flaherty KR (2020). Pamrevlumab, an anti-connective tissue growth factor therapy, for idiopathic pulmonary fibrosis (PRAISE): a phase 2, randomised, double-blind, placebo-controlled trial. Lancet Respir Med.

[35] Ramazani Y, Knops N, Elmonem MA, Nguyen TQ, Arcolino FO, van den Heuvel L (2018). Connective tissue growth factor (CTGF) from basics to clinics. Matrix Biol.

[36] Sterneck E, Poria DK, Balamurugan K (2020). Slug and E-cadherin: stealth accomplices?. Front Mol Biosci.

[37] Su J, Morgani SM, David CJ, Wang Q, Er EE, Huang YH (2020). TGF-β orchestrates fibrogenic and developmental EMTs via the RAS effector RREB1. Nature.

[38] Nagai K, Arai H, Okudera M, Yamamura T, Oki H, Komiyama K (2014). Epiregulin is critical for the acinar cell regeneration of the submandibular gland in a mouse duct ligation model. J Oral Pathol Med.

[39] Enger TB, Samad-Zadeh A, Bouchie MP, Skarstein K, Galtung HK, Mera T (2013). The Hippo signaling pathway is required for salivary gland development and its dysregulation is associated with Sjogren’s syndrome. Lab Invest.

[40] Aure MH, Symonds JM, Mays JW, Hoffman MP (2019). Epithelial cell lineage and signaling in murine salivary glands. J Dent Res.

[41] Knox SM, Lombaert IM, Reed X, Vitale-Cross L, Gutkind JS, Hoffman MP (2010). Parasympathetic innervation maintains epithelial progenitor cells during salivary organogenesis. Science (New York, NY).

[42] Hauser BR, Aure MH, Kelly MC, Hoffman MP, Chibly AM (2020). Generation of a single-cell RNAseq atlas of murine salivary gland development. J iScience..

[43] Song EC, Min S, Oyelakin A, Smalley K, Bard JE, Liao L (2018). Genetic and scRNA-seq analysis reveals distinct cell populations that contribute to salivary gland development and maintenance. Sci Rep.

[44] Sekiguchi R, Martin D, Yamada KM (2020). Single-cell RNA-seq identifies cell diversity in embryonic salivary glands. J Dent Res.

[45] Jung DW, Hecht D, Ho SW, O'Connell BC, Kleinman HK, Hoffman MP (2000). PKC and ERK1/2 regulate amylase promoter activity during differentiation of a salivary gland cell line. J Cell Physiol.

[46] Azen EA, Latreille P, Niece RL (1993). PRBI gene variants coding for length and null polymorphisms among human salivary Ps, PmF, PmS, and Pe proline-rich proteins (PRPs). Am J Hum Genet.

[47] Jurisic V, Radenkovic S, Konjevic G (2015). The actual role of LDH as tumor marker, biochemical and clinical aspects. Adv Exp Med Biol.

[48] Hai B, Zhao Q, Deveau MA, Liu F (2018). Delivery of sonic hedgehog gene repressed irradiation-induced cellular senescence in salivary glands by promoting DNA repair and reducing oxidative stress. Theranostics.

[49] Sanz-Ramos P, Dotor J, Izal-Azcárate I (2014). The role of Alk-1 and Alk-5 in the mechanosensing of chondrocytes. Cell Mol Biol Lett.

[50] Li L, Wang J, Chau JF, Liu H, Li B, Hao A (2013). Smad1 stabilization and delocalization in response to the blockade of BMP activity. Cell Mol Biol Lett.

[51] Zhang CY, Mao L, Li L, Tian Z, Zhou XJ, Zhang ZY (2007). Promoter methylation as a common mechanism for inactivating E-cadherin in human salivary gland adenoid cystic carcinoma. Cancer.

[52] Wong KY, Chim CS (2015). DNA methylation of tumor suppressor protein-coding and non-coding genes in multiple myeloma. Epigenomics.

