# Supplementary material for: Reversely immortalized mouse salivary gland cells presented a promising metabolic and fibrotic response upon BMP9/Gdf2 stimulation
Source: Cell Mol Biol Lett. 2022 Jun 11;27:46. doi: 10.1186/s11658-022-00333-9 (PMC9188258; doi:10.1186/s11658-022-00333-9)
Supplement: Supplementary file 1 — Additional file 1: Figure S1. Flow cytometry analysis of the bare expression of Krt7 and Krt19 in iSMGCs or SLGCs. Figure S2. Immunofluorescence staining exhibited positive expression of BMP9 in ducts and mesenchyme in SMG and SLG at four and eight weeks postnatal. Bar = 75 μm. Figure S3. Upregulation of Bmp9 and Col1a1 in response to submandibular gland ductal ligation. (A) Gross morphology of submandibular gland after a seven day duct ligation. (B) histomorphology of SMG after a seven day duct ligation were examined by H&E staining. (C) The mRNA expression of Bmp9 and Col1a1 were increased after a seven day duct ligation and detected by RT-qPCR. All experiments were repeated at least three times and compared with a control. **P < 0.01, ***P < 0.001. [file 11658_2022_333_MOESM1_ESM.docx]

**Supplementary Figures and Legends**


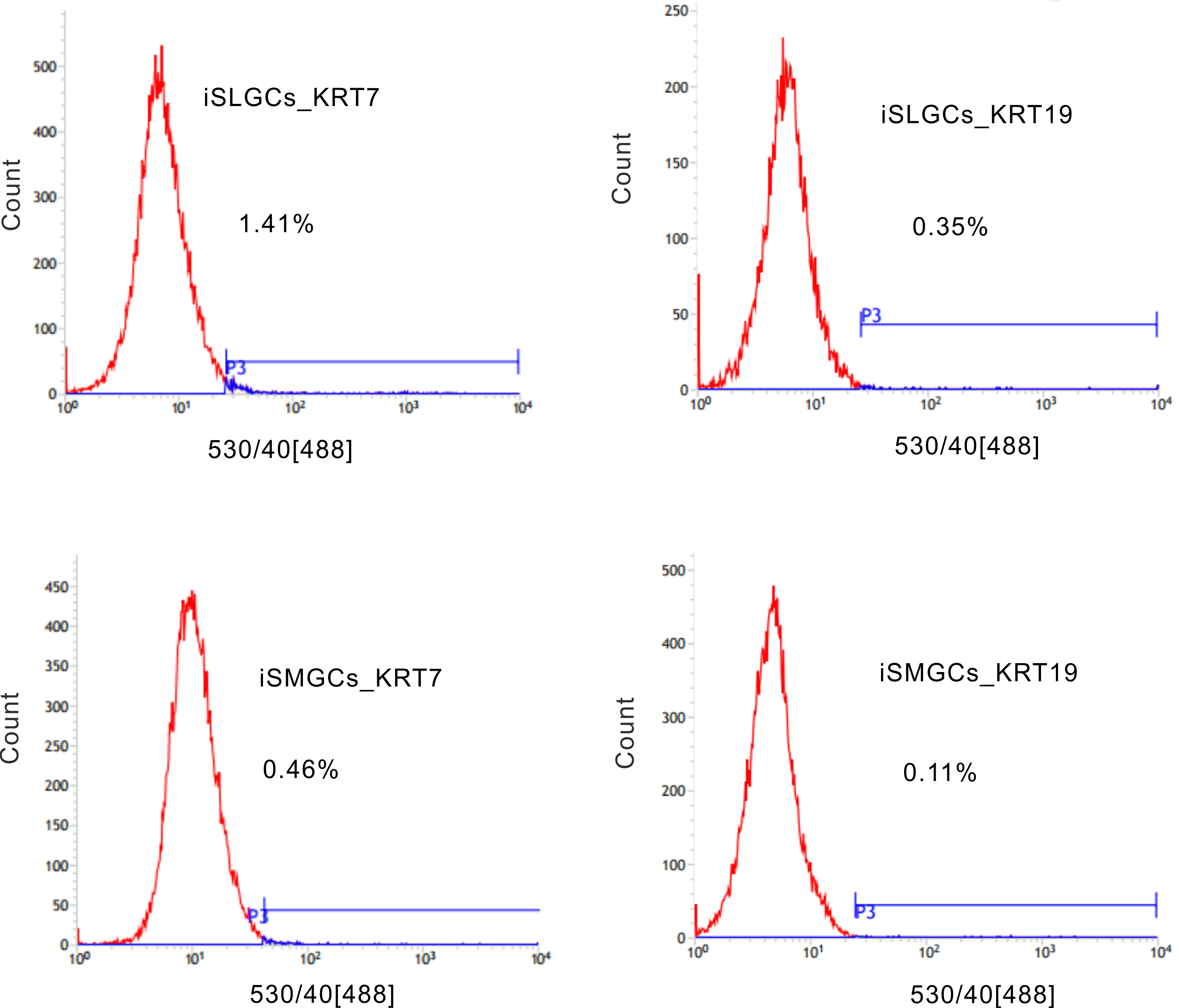


Figure S1. Flow cytometry analysis of the bare expression of KRT7 and KRT19 in iSMGCs or iSLGCs.


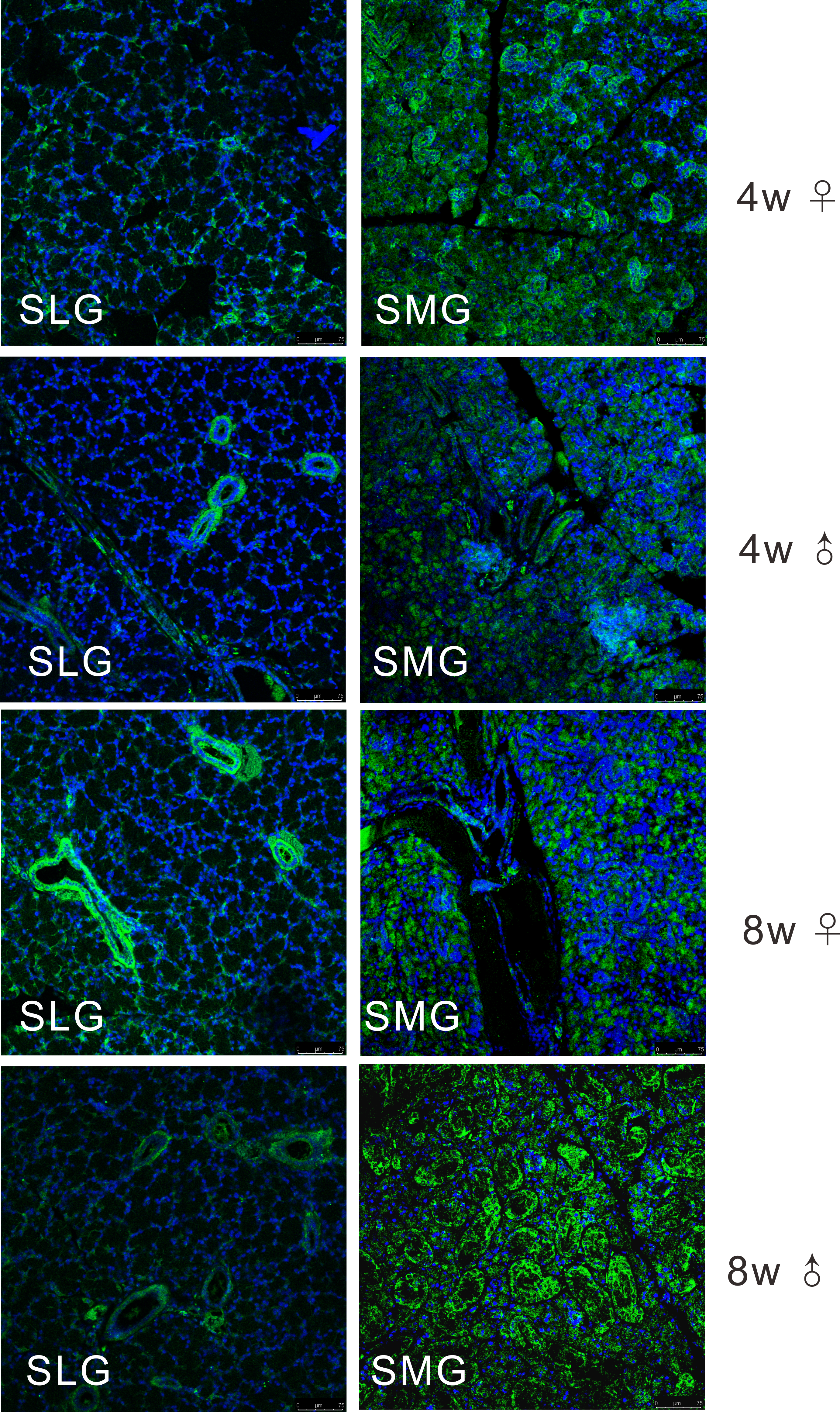


Figure S2. Immunofluorescence staining exhibited positive expression of BMP9 in ducts and mesenchyme in SMG and SLG at four and eight weeks postnatal. Bar=75 μm.


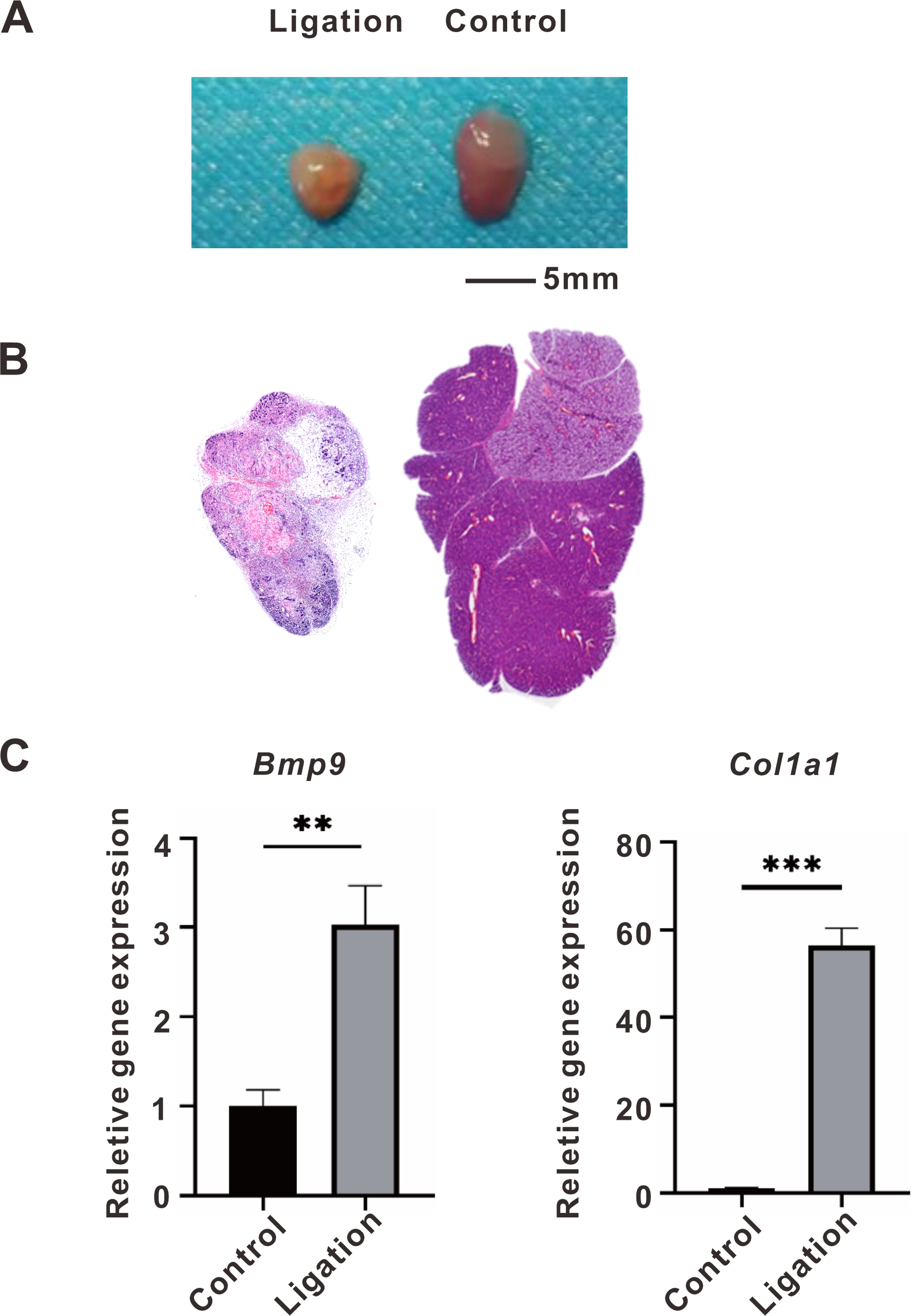


Figure S3. Upregulation of *Bmp9*and *Col1a1* in response to submandibular gland ductal ligation. (A) Gross morphology of submandibular gland after a seven day duct ligation. (B) histomorphology of SMG after a seven day duct ligation were examined by H&E staining. (C) The mRNA expression of *Bmp9* and *Col1a1* were increased after a seven day duct ligation and detected by RT-qPCR. All experiments were repeated at least three times and compared with a control. **, P＜0.01, ***, P＜0.001.
